# Ozone Therapy versus Hyaluronic Acid Injections for Pain Relief in Patients with Knee Osteoarthritis: Preliminary Findings on Molecular and Clinical Outcomes from a Randomized Controlled Trial

**DOI:** 10.3390/ijms24108788

**Published:** 2023-05-15

**Authors:** Cristiano Sconza, Berardo Di Matteo, Paolo Queirazza, Arianna Dina, Roberta Amenta, Stefano Respizzi, Giuseppe Massazza, Antonio Ammendolia, Elizaveta Kon, Alessandro de Sire

**Affiliations:** 1 Department of Biomedical Sciences, Humanitas University, 20072 Milan, Italy; cristiano.sconza@humanitas.it (C.S.); paolo.queirazza@humanitas.it (P.Q.);; 2 IRCCS Humanitas Research Hospital, 20089 Milan, Italy; 3 Physical Medicine and Rehabilitation, University of Milan, 20122 Milan, Italy; 4 Department of Rehabilitation, Casa di Cura Villa Aurelia, 96100 Syracuse, Italy; 5 Division of Physical Medicine and Rehabilitation, Department of Surgical Sciences, University of Turin, 10124 Turin, Italy; 6 Department of Medical and Surgical Sciences, University of Catanzaro “Magna Graecia”, 88100 Catanzaro, Italy

**Keywords:** ozone, ozone treatment, hyaluronic acid, intra-articular injections, osteoarthritis, knee osteoarthritis

## Abstract

Ozone therapy (OT) is used for the treatment of multiple musculoskeletal disorders. In recent years, there has been a growing interest in its use for the treatment of osteoarthritis (OA). The aim of this double-blind randomized controlled trial was to evaluate the efficacy of OT compared with hyaluronic acid (HA) injections for pain relief in patients with knee OA. Patients with knee OA for at least three months were included and randomly assigned to receive three intra-articular injections of ozone or HA (once a week). Patients were assessed at baseline and at 1, 3, and 6 months after the injections for pain, stiffness, and function using the WOMAC LK 3.1, the NRS, and the KOOS questionnaire. Out of 55 patients assessed for eligibility, 52 participants were admitted to the study and randomly assigned into the 2 groups of treatment. During the study, eight patients dropped out. Thus, a total of 44 patients, reached the endpoint of the study at 6 months. Both Group A and B consisted of 22 patients. At 1-month follow-up after injections, both treatment groups improved statistically significantly from baseline in all outcomes measured. At 3 months, improvements remained similarly consistent for Group A and Group B. At 6-month follow-up, the outcomes were comparable between the 2 groups, showing only a worsening trend in pain. No significant differences were found between the two groups in pain scores. Both therapies have proven to be safe, with the few recorded adverse events being mild and self-limiting. OT has demonstrated similar results to HA injections, proving to be a safe approach with significant effects on pain control in patients affected by knee OA. Due to its anti-inflammatory and analgesic effects, ozone might be considered as a potential treatment for OA.

## 1. Introduction

Osteoarthritis (OA) of the knee is a widespread degenerative disease that causes pain, joint stiffness, and reduced motor function [1,2,3]. Recent estimates indicate that there are currently about 654 million individuals (40 years and older) with knee OA [4]. Studies conducted in several countries have shown that the prevalence of knee OA is higher among individuals with lower socioeconomic status and in females. The ratios of prevalence and incidence in females to males were 1.69 and 1.39, respectively [5,6]. The resulting disability increases the risk of psychological distress and reduces the quality of life for millions of people worldwide [7,8,9]. Chronic exposure to low-grade inflammation and an imbalance in oxidant–antioxidant systems seem to play a role in the pathogenesis of OA, mostly impairing the regulation of cartilage, thus making necessary an early diagnosis of this disease [10,11]. A crucial role in the pathogenic process could be played by the overexpression of inflammatory cytokines released by chondrocytes (IL-1, IL-6, IL-8, IL-17, TNF-α, IFN-γ), which promote cartilage catabolism [12]. Inflammatory cytokines can also generate overproduction of ROS (reactive oxygen species) by increasing cellular oxidative stress and resulting in the activation of the NF-Kβ pathway, which leads to accelerated cartilage matrix disintegration and apoptosis [13,14].

There are currently no approved treatments that can slow OA-related structural progression, so the main goals of conservative treatment are to provide symptomatic pain relief, improve joint function, and delay surgery [8,11,13]. The conservative approach to KOA consists of numerous treatments ranging from everyday living adjustments (the use of a cane, insoles, patient education, weight loss) to oral analgesic drugs such as nonsteroidal anti-inflammatory drugs (NSAIDs), physical therapy (aerobic, proprioceptive, and strengthening training), and instrumental physical modalities [15,16,17].

Several minimally invasive strategies, such as intra-articular (IA) injections, have been shown to be well tolerated and able to provide good clinical results [18,19,20]. IA drug delivery is a method that allows high local bioavailability with low systemic exposure, fewer adverse events, and reduced costs [21,22].

These treatments are of particular interest because they could provide specific actions on the pathogenic biochemical mechanisms previously described. Hyaluronic acid (HA) injections are known to improve mechanical properties of the joint such as shock absorption, lubrication, and cartilage protection [23]. HA has been shown to possess chondroprotective, anti-inflammatory, and lubricating action on the joint [24]. The beneficial effect on cartilage is due to the binding between HA and CD44 which reduces apoptosis of chondrocytes while stimulating their proliferation and inhibits the expression of interleukin (IL)-1β with a consequent reduction in the release of several matrix metalloproteinases (MMP 1, 2, 3, 9, and 13) and an anti-inflammatory effect. In addition, due to its physical properties and viscosity, HA can lubricate the joint, decreasing its degeneration caused by friction, which is related to the role that collagen might play for synovial fibroblasts [24,25]. Indeed, the use of collagen has been growing in recent years not only for the treatment of nerve injuries [26] but also the management of OA with potential beneficial effects of its supplementation in terms of pain relief and improvement of function [27].

Furthermore, recent studies have shown that HA may also have a regenerative role at the bone level. HA appears to be able to promote an enhancement of the osteogenetic effect of BMP-2 through the activation of a pathway involving the Smad family of proteins and the downregulation of certain BMP-2 antagonists (noggin and follistatin) [28,29,30].

There is a large body of evidence that has confirmed HA efficacy in the management of knee OA [31,32,33,34,35]. Indeed, in recent years, there has also been a growing interest in the effects of IA injections of ozone (O3) for the treatment of knee OA. The strong interest of the scientific community is evidenced by the growing number of published articles showing promising results of ozone therapy (OT), although a poor methodological quality has been reported by recent systematic reviews [36,37,38,39]. In this context, a lack of valuable scientific studies prevents an accurate assessment of the efficacy of OT in the pain management of KOA patients [36,37,38].

OT is commonly used in the management of a variety of musculoskeletal disorders, mainly for its analgesic, anti-inflammatory, immunomodulatory, and trophic properties [40,41,42]. The rationale behind the medical use of O3 lies in its intrinsic chemical properties: it has been shown that, once injected into a joint capsule, O3 is able to induce an acute moderate–intensive oxidative stress throughout the generation of ROS and lipid oxygen products (LOPs) [42]. In response, the endogenous antioxidant system is stimulated, resulting in an overall immunomodulation via the upregulation of anti-inflammatory cytokines (IL-4, IL-10), growth factors (TGF-ß, IGF-1), and antioxidant enzymes (superoxide dismutase, catalase, glutathione peroxidase) versus the downregulation of inflammatory cytokines (IL-1ß, IL-6, TNF alpha, COX-2) and proteolytic enzymes [43,44]. Through the regulation of these factors, OT creates an environment that counteracts the proinflammatory and pro-oxidative circuits present in knee OA.

The aim of the present double-blind randomized controlled trial (RCT) was to compare the clinical outcomes at 6 months follow-up of IA OT versus HA injections for the treatment of knee OA. Our hypothesis was that OT might provide symptomatic relief and functional improvement at least comparable to that of HA.

## 2. Results

A total of 55 patients were assessed for eligibility, 3 of whom were excluded (2 did not meet the inclusion criteria, and 1 declined to participate). Thus, the remaining 52 participants were admitted to the study and randomly assigned into the 2 groups of treatment.

During the study, eight patients dropped out: three who voluntarily decided to discontinue the treatment (one assigned to O3 group, two to the HA group) and five who decided to undergo other analgesic treatments or did not complete the scheduled follow-up (three assigned to O3 group and two to the HA group).

**Figure 1:**
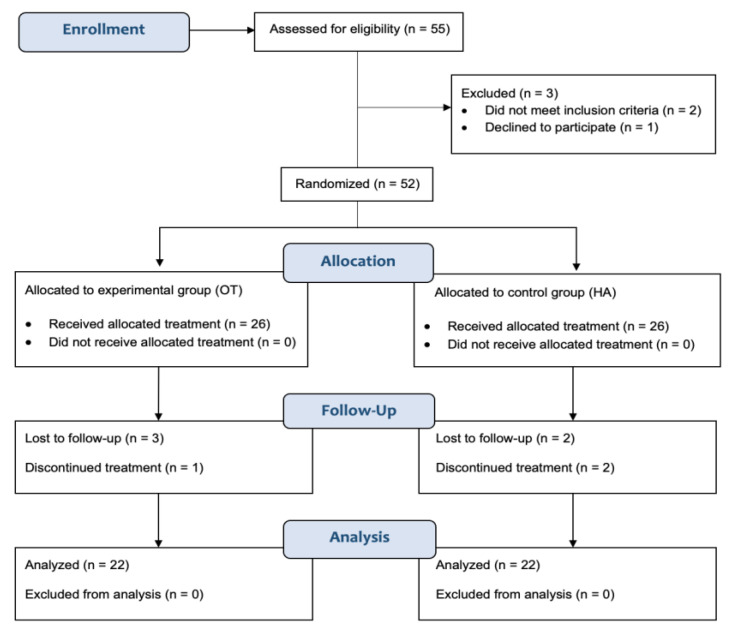
PRISMA Flow Diagram.

Therefore, a total population of 44 participants, randomized into 2 groups, reached the endpoint of the study at 6 months (see PRISMA Flow Diagram depicted in Figure 1 for further details).

Group A consisted of 22 patients (12 male and 10 female) and received OT. Group B consisted of 22 patients (8 male and 14 female) and underwent HA injections (Group B). The baseline characteristics of the patients enrolled and those who completed the study are presented in Table 1.

The groups were homogeneous in terms of gender, age, body mass index (BMI), and previous treatment received. No statistically significant difference was observed in the baseline outcome measures.

Changes in clinical scores recorded during the follow-up period for the two groups are presented in Table 2. Data are reported on patients who completed follow-up (per-protocol analysis). We have also included the analysis of all recruited patients (intention-to-treat analysis) in Supplementary File S1. The results of both analyses showed no significant differences.

### 2.1. Western Ontario and McMaster Universities Osteoarthritis Index Pain Subscore

**Figure 2:**
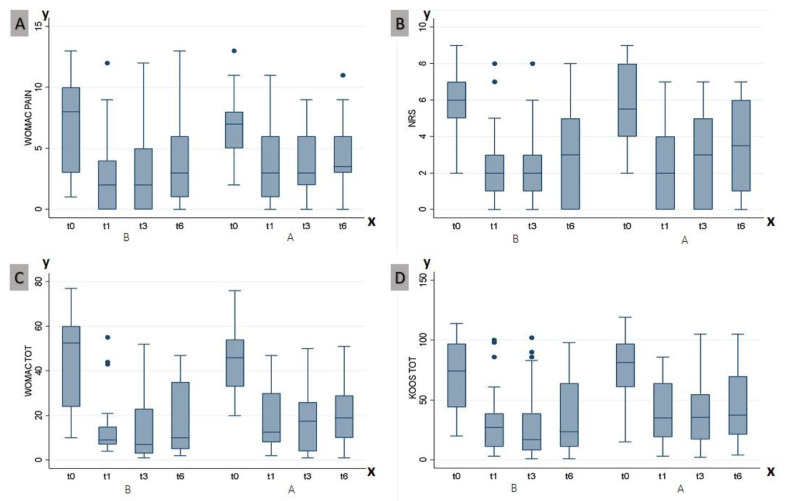
Intra-group analysis of the WOMAC LK 3.1 pain subscale score (**A**), NRS (**B**), WOMAC total score (**C**), and KOOS (**D**) in the groups of ozone (**A**) and HA (**B**). On the X axis are the above outcome measures; on the Y axis are the time intervals of the study: baseline (T0), 1 month (T1), 3 months (T3), and 6 months (T6) after treatment. Abbreviations: WOMAC: Western Ontario and McMaster Universities Osteoarthritis Index; KOOS: Knee injury and Osteoarthritis Outcome Score; NRS: Numeric Rating Scale.

Data analysis showed a significant increase in both groups in the Western Ontario and McMaster Universities Osteoarthritis Index (WOMAC) pain (WOMACp) subscore. At baseline, the mean score of WOMACp was 6.8 in Group A and 7.3 in Group B. After reassessment at 1-month follow-up, it was 3.7 ± 3.1 in Group A and 2.8 ± 3.1 in Group B. A similar trend was found in the following evaluations: at 3 months follow-up the score for Group A was 3.7 ± 2.7, while in Group B it was 2.9 ± 3.1; at 6 months follow-up it was 4.4 ± 3.1 in Group A and 3.9 ± 3.8 in Group B (see Figure 2).

Both treatment groups showed a comparable improvement in the WOMAC pain subscale. The interaction effects of group and time were statistically significant on the outcome (*p* = 0.001). No significant differences were observed between groups at the various time intervals (Table 3).

### 2.2. Numeric Rating Scale

Pain assessment using the Numeric Rating Scale (NRS) scale showed similar values in the two groups at the first assessment. At baseline, the NRS score was 5.6 ± 2.2 in Group A and 5.8 ± 1.7 in Group B. In both groups, an improvement in pain was recorded after treatment: at 1 month, the NRS score was 2.3 ± 2.2 in Group A and 2.6 ± 2.2 in Group B; at 3 months 2.9 ± 2.6 and 2.5 ± 2.3, respectively; and at 6 months 3.6 ± 2.6 and 3.2 ± 2.7, respectively (as depicted in Figure 2).

The interaction effects of group and time were statistically significant on the outcome (*p* < 0.001). No significant differences were observed in the intra-group analysis at various time intervals (as shown in Table 4).

### 2.3. Western Ontario and McMaster Universities Osteoarthritis Index Questionnaire

The mean WOMAC score at baseline was comparable in the 2 groups, with no statistical differences between Group A and Group B, at 45.6 ± 15.1 and 45.8 ± 20.1, respectively. An improvement in the score at 1-, 3-, and 6-month follow-up was recorded for both groups: at 1-month follow-up it was 18.7 ± 14.3 in Group A and 14.0 ± 13.1 in Group B; at 3 months follow-up it was 17.7 ± 14.0 and 14.8 ± 16.4, respectively; and at 6 months it was 20.9 ± 15.8 and 17.9 ± 16.7 (see Figure 2). Both treatment groups showed a comparable improvement in the WOMAC scale.

The interaction effects of group and time were statistically significant on the outcome (*p* < 0.001). No significant differences were observed between groups at the various time intervals (see Table 4 for further details).

### 2.4. Knee Injury and Osteoarthritis Outcome Score

No significant difference was found at baseline after the KOOS score assessment: 79.1 ± 24.9 in Group A and 72.2 ± 27.3 in Group B. An improvement in the score was recorded in the subsequent assessments for Group A and Group B: at 1-month follow-up it was 40.0 ± 26.2 and 32.4 ± 26.9, at 3 months it was 38.9 ± 28.1 and 30.9 ± 32.8, and at 6 months it was 44.8 ± 30.5 and 35.8 ± 32.3, respectively (see Figure 2).

The interaction effects of group and time were statistically significant on the outcome (*p* < 0.001). No significant inter-group differences were observed at the various time intervals (Table 4).

### 2.5. Safety

Both therapies proved to be safe; the few adverse events recorded were mild and self-limiting until 24 h after injections. Patients treated with OT reported adverse events in 11% of cases. The most reported symptom was the transient sensation of swelling immediately after the procedure and for a few hours thereafter. In the HA group, 10% of patients complained of self-limiting pain and a swollen feeling after injections.

## 3. Discussion

According to our results, treatments with OT and HA injections are able to produce similar positive results in patients’ pain control and function recovery. Although these data are preliminary and need to be confirmed on a larger study population, they raise some interesting considerations. Both groups improved with statistical significance over baseline in all outcomes measured from 1-month post-treatment and remained stable, showing only a trend toward slight worsening up to 6 months. No significant differences were found between the two treatments in pain scores. Concerning scores regarding function and quality of life, although without statistical significance HA seems to show a slightly better trend at 3 months (KOOS) and 6 months follow-up (KOOS and WOMAC tot). Both therapies appeared to be safe: the few adverse events recorded were mild and self-limiting.

Ozone gas was discovered in 1840, and its expansion into the medical field has given rise to exciting research in recent decades to validate its clinical value [45]. In recent years, more and more attention has been paid to its IA use due to its capacity to modulate inflammation with potential protective effects on cartilage and reduction in oxidative stress [33,36,46,47,48]. In synovial fluid, O3 may be able to reduce the production of proinflammatory cytokines, particularly IL-6, IL-1β, and TNF-α, which are responsible for cartilage degradation, [49,50] improving serum IGF-1 levels [51]. IGF-1 is a growth factor with important properties in reducing inflammation and stimulating cell growth, differentiation, and tissue repair [52,53,54,55]. O3 could also act to reduce the NF-Kβ activation pathway and enhance the Nrf2 (nuclear factor erythroid 2-related factor 2) pathway [56,57]. The activated NF-Kβ pathway could lead to the cascade activation of other proinflammatory cytokines; therefore, its inhibition would reduce cartilage matrix degradation and initiation of the apoptotic pathway, thereby promoting cell survival [57,58]. In contrast, Nrf2 activation by small and repeated oxidative stresses is linked to the generation of antioxidant response elements (AREs) [59,60,61]. This could result in an improved response to pathological radical stress, which is common to most chronic inflammatory diseases [60,61,62,63]. The molecular aspects listed above may therefore support the positive results shown in our study for O3 treatment. In particular, the possibility of reduced serum levels of inflammatory mediators could explain the rapid reduction in pain experienced by patients (as early as 1 month after the procedure).

Looking at the available literature, an overall modest methodological quality has been underlined by recent systematic reviews and meta-analyses [36,37,38]. Indeed, only a few RCTs comparing OT with other injectables are available, and only a fraction of them present a double-blinded design and a proper adherence to the CONSORT guidelines for reporting results [36,37,38]. Furthermore, relevant discrepancies in O3 therapeutic protocols have been documented [13,46]. Based on these findings, the present trial was conceived with a double-blinded design, including a clear description of the randomization method, and by adopting a therapeutic protocol established by the International Scientific Committee of Ozone Therapy (ISCO3) [64].

On the other hand, HA has already a large body of evidence on its safety and efficacy as a conservative treatment of knee OA, due to its mechanical and viscoelastic properties of shock absorption and improvement of joint lubrication, as well as its anti-inflammatory and cartilage-protective actions [32,65,66]. For this reason, we chose to evaluate the outcomes of O3 treatment against a strong comparator such as HA. O3 has already been tested against a placebo [67], showing better results in terms of knee pain control and improvement of motor function, and compared with a steroid [51,68,69,70]. In the latter study, the authors noted a significant improvement in clinical scores but also a reduction in serum levels of inflammatory cytokines, especially IL-1b and TNF-α, after O3 injections. These levels were lower than those of patients treated with steroids after 2 and 6 months, demonstrating the possible long-lasting anti-inflammatory effect of O3 [51].

From what has been discussed so far, the positive result that O3 and HA achieved on pain control in our study is understandable. What is more complex, and certainly requires confirmation on larger case series, is to explain the rapid positive functional recovery achieved at WOMAC and KOOS scores. Usually, better control of pain symptoms can lead to a progressive and slower improvement in motor and functional abilities. One explanation for this could be related to the different etiopathogenetic hypotheses related to knee osteoarthritis and consequently to the possibility that one type is more present in our limited study sample. In fact, both O3 and HA have different mechanisms of action that may be effective for the treatment of knee OA [13,24,25]. This is relevant considering that OA is not only a degenerative disease, rather both mechanical and inflammatory factors are attributed to its pathophysiology [10,11]. Notably, RNA-Seq analysis of knee OA cartilage suggested the presence of two major pathogenic pathways mirroring two OA phenotypes: one more inflammatory and one non-inflammatory and more load-related [71,72]. Proper classification may be relevant to treatment choice: O3, HA, and other available therapies target one or a few molecular mechanisms, so they may not be equally effective for all phenotypes. This could explain why, despite the results of our study, the comparison of the efficacy between the two treatments still presents controversial results.

In recent years, several studies have already tried to compare the effectiveness of O3 and HA in the treatment of knee OA [73,74,75,76,77]. Both treatments proved to be effective in the management of pain and other OA-related symptoms; however, the results were conflicting regarding the duration and impact of the approaches.

In more detail, in 2016 Giombini et al. compared the IA injection of O3, HA, and a combined therapy of both [77]. They reported significant (*p* < 0.05) pain reduction and disability with all 3 approaches (OT, HA, and their combination) at the end of the treatment cycle and at the 2-month follow-up evaluation. Moreover, the combination of OT and HA treatment led to a significantly better outcome, especially after two months [77]. Invernizzi et al. showed that OT was responsible for faster pain reduction, whereas HA showed longer lasting efficacy [73]. Raeissadat et al. studied a cohort of 174 patients with knee pain for at least 3 months and treated with a cycle of injections comparable to ours in number (3 injections every week) but with a higher O3 dosage (10 mL at a concentration of 30 μg/mL) [75]. Patients improved on all outcome measures with comparable results between the two groups. These data therefore seem to agree with our results but highlight one of the problems related to the different therapeutic protocols of administering IA OT, and the consequent difficulty in comparing the results of multiple trials, as evidenced also in previous studies [36,37,38]. Another aspect that makes the comparison complex is the specific HA used: with current technological advances, there are many varieties of HA with different therapeutic effects (linear, cross-linked, high/low molecular weight, HA associated with other molecules). In 2020, de Sire et al. compared the long-term effects of OT versus HA [76]. This trial was conducted on 42 patients affected by knee OA who received weekly injections of O3 or HA and periodic follow-up visits up to 31 weeks after treatment. The results revealed that OT was comparable to HA in terms of pain reduction in the long term, but at 1-month follow-up HA evidenced better results [76].

The present study has several limitations. First, the small sample size of the analyzed cohort prevents robust data analysis on the efficacy of OT compared with HA. Another limitation of the study is that we were unable to draw definitive conclusions about the long-term effects of the treatment. Furthermore, no radiographic or biochemical data are available for comparison. Future prospects lay in performing studies that can confirm these preliminary results on a larger cohort of patients, evaluated at a longer follow-up (at least 1 year), by also using imaging data as well as biochemical parameters (e.g., the level of inflammatory cytokines in synovial fluid or blood).

## 4. Materials and Methods

### 4.1. Participants

Knee OA patients were recruited from the Department of Rehabilitation and Functional Recovery of the Humanitas Research Hospital, Rozzano, Milan, Italy from January 2021 to March 2022.

An experienced rehabilitation physician screened the patients for eligibility by evaluating the following inclusion criteria: (1) symptomatic unilateral knee with history of chronic pain (at least 3 months) and resistant to at least 1 attempt at conservative treatment (e.g., physical therapy, analgesics); (2) pain score measured with the WOMAC LK 3.1 subscale between ≥9 and ≤19; (3) X-ray imaging findings of osteoarthritis (Kellgren–Lawrence score of 2–3).

Exclusion criteria were: (1) age younger than 18 years and older than 80 years; (2) BMI greater than 40 kg/m^2^; (3) presence of active knee infection or inflammatory arthropathy; (4) knee ligament injury or knee replacement; (5) knee surgery in the previous year; (6) presence of systemic disorders such as diabetes, rheumatologic disorders, hematologic diseases (coagulopathy), severe cardiovascular disease, immunodepression; (7) cognitive impairment or psychiatric disease; (8) drug or alcohol dependence; (9) pregnancy or lactation status; (10) previous (3 months) IA injection therapy.

### 4.2. Study Design

This RCT was approved by the Hospital Ethics Committee and Scientific Board (Authorization n° 2556, 27 May 2020). The study protocol was registered on clinicaltrials.gov (NCT04426721). The study was performed in accordance with the Ethical Principles for Medical Research Involving Human Subjects outlined in the Declaration of Helsinki. All participants were fully informed about all experimental procedures and signed a written informed consent form prior to participation.

A total of 52 patients were randomly divided into 2 different treatment groups: those receiving 3 weekly IA injections of O3 (Group A) versus those receiving 3 weekly administrations of high-molecular-weight HA (Group B).

The randomization list (block randomization with block sizes of 8 patients) was provided by an independent statistician and kept in a dedicated office. Progressively numbered, sealed envelopes containing the treatment allocation (OT or HA) were used. The physician administering the treatment contacted the office just before performing the injection to know the patient allocation; the envelope was then opened to determine the treatment group and the patients included in the randomization list. Before the injection, the syringe was appropriately covered to prevent patients from discovering the substance they were receiving. The treatment consisted of 3 injections at 1-week intervals. Patients were then prospectively evaluated at baseline and at 1, 3, and 6 months after the injections. At the end of the study, the nature of the injected substance was revealed to the patients.

### 4.3. Intervention

A physician of our institute with expertise in IA injections performed the treatment according to the following technique. In both groups, 32 mm (22 G) sterile needles were used. We performed IA injections with a masked syringe on supine patients with the knee flexed at 90, in sterile conditions (skin cleanse and double disinfection with iodopovidone 7.5%). The injection approach performed was anterior and lateral to the patellar tendon and between the inferior margin of the lateral femoral condyle and the superior margin of the tibial plateau. No pre-medication or anesthesia were used.

#### 4.3.1. Ozone Therapy

Patients of Group A received an IA injection of a mixture of oxygen–ozone (total of 10 mL) with an O3 concentration of 10 μg/mL, obtained by means of a Multiossigen Medical 99 IR generator (Multiossigen s.r.l., Gorle, Bergamo, Italy). The machine converted medical oxygen into a mixture of O3 (0.05%) and O2 (99.95%) through an electrochemical process. It was equipped with a photometer, calibrated according to the standard iodometric titration of O3, and a voltage system that regulated the concentration.

#### 4.3.2. Hyaluronic Acid Infiltrations

Patients of Group B received injections of HA (Sinovial^®^, IBSA Farmaceutici, Lodi, Italy). The viscous solution was injected into the knee joint using the same approach as for Group A. Sinovial^®^ (sodium hyaluronate) is a non-modified HA, a linear polymer composed of the disaccharide units N-acetyl-d-glucosamine and Na-d-glucuronate, linked by glycosidic bonds. It is classified as a medical device; it is a 0.8% physiological solution of HA (32 mg/2 mL) in sodium chloride in a ready-to-use sterile syringe for IA injection. HA is obtained by biofermentation and undergoes a stringent purification process (in the absence of chemical modification) to produce a highly purified, non-pyrogenic polymer of a defined molecular weight (800–1200 kDa) that is completely free from animal proteins.

After the injection, patients were sent home with instructions to restrict the use of the leg for at least 24 h and to use ice or other cold therapy on the affected area to relieve pain. During the treatment period, rest or mild activities were permitted, and subsequently a gradual resumption of normal sport or recreational activities was allowed as tolerated.

### 4.4. Outcome Measures

Patients were prospectively evaluated at baseline and then at 1, 3, and 6 months after the last injection. As the primary outcome was to compare the effectiveness of the two treatments, changes in knee pain were investigated through the WOMAC using the Likert scale, Version 3.1 (WOMAC LK 3.1)—pain subscale [78]. In addition, we considered the following secondary outcome measures: WOMAC LK 3.1, KOOS [79], and NRS [80].

The WOMAC LK 3.1 is a validated tool used for assessing knee pain, stiffness, and function. It is composed of 24 items: 5 assessing knee pain, 2 assessing knee stiffness, and 17 assessing physical function. Each item is answered on a 5-point Likert scale, with grading from 0 (none or never) to 4 (extreme or always). A higher score indicates worse pain, stiffness, or functional limitation. NRS is a validated measure of knee pain. It is an 11-point Likert-type scale anchored by 0 “no pain” and 10 “worst possible pain”. Subjects rate their average pain over the last 48 h. The Knee injury and Osteoarthritis Outcome Score (KOOS) is a knee-specific instrument developed to assess the patient’s opinion about their knee and associated problems. It holds 42 items in 5 separately scored subscales; Pain, other Symptoms, Function in daily living (ADL); Function in Sport and Recreation (Sport/Rec); and knee-related Quality of Life (QOL).

Lastly, we assessed the safety by considering the number of adverse events. To guarantee the double-blinding of the trial, all the clinical evaluations were performed by an independent physician not involved in the injection procedure.

### 4.5. Statistical Analysis

The statistical analysis of the collected data was performed using the statistical package R 3.5.2 (R foundation, Vienna, Austria). Descriptive analyses were generated for the demographic and clinical variables of the two arms.

Categorical variables were summarized using frequency and percentage. Continuous variables were summarized using mean, median standard deviation, minimum, and maximum. To assure that randomization was successful, the following tests were conducted: (1) age and baseline WOMAC Pain comparisons of OT and HA groups by using two-tailed independent sample *t*-tests, (2) gender distribution by using a Fisher’s Exact test, and (3) race distribution by using a Likelihood-Ratio Chi-square test. Each of these 3 tests was conducted using alpha = 0.05.

The primary outcome of this study was the change in the WOMAC LK 3.1 pain score (from baseline to 6 months post-injection). A 2-tailed independent sample *t*-test was used to test this endpoint using alpha = 0.05. If any critical baseline variables (i.e., age, gender, BMI, race, or baseline WOMAC Pain) were found to differ significantly between treatment groups, then an ANCOVA (Analysis of Covariance) was performed in place of a *t*-test. Only variables that differed between groups were entered into the model. Secondary objectives of this study included determining whether OT was superior to HA injections with regard to the improvement in NRS Pain, KOOS, and the improvement in WOMAC Pain and Function.

Subject-reported outcome measures: WOMAC LK 3.1, KOOS, and NRS Pain were summarized and thoroughly characterized with appropriate descriptive statistics including error measures. Categorical variables were summarized using frequency and percentage. Continuous variables were summarized using mean, median standard deviation, minimum, and maximum.

The analysis was performed only on patients who completed follow-up, using a per-protocol analysis method. We also included the analysis performed on all recruited patients, according to the intention-to-treat analysis method.

## 5. Conclusions

This study suggested that treatment with OT and HA injections could produce similar positive results in pain control and function recovery in patients with knee OA. Although these data are preliminary and must be verified on a larger study population, both therapies have shown interesting properties in terms of safety and efficacy.

Further studies with a larger sample size and longer follow-up are needed to understand which types of patients could benefit more from one approach compared with the other.

## Figures and Tables

**Table 1:** Baseline characteristics of study participants.

	OT Group (Group A)	HA Group (Group B)	*p* Value
*Included*			
Number of patients	26	26	
Sex (male)	15 (54.7%)	10 (38.5%)	0.18
Age (years)	68.0 ± 10.1 (min: 48; max: 79)	67.96 ± 9.6 (min: 49; max: 79)	1.00
BMI (kg/m^2^)	29.08 ± 4.68	27.87 ± 5.02	0.39
*Analyzed*			
Number of patients	22	22	
Sex (male)	12 (54.6%)	8 (36.4%)	0.36
Age (years)	68.5 ± 9.1 (min: 48; max: 79)	69.1 ± 8.7 (min: 50; max: 79)	0.86
BMI (kg/m^2^)	28.65 ± 4.57	28.13 ± 5.12	0.72

Continuous variables are expressed as means ± standard deviations; categorical data are expressed as counts and percentages. Abbreviations: OT: oxygen–ozone therapy; HA: hyaluronic acid; BMI: body mass index.

**Table 2:** Intra-group differences in outcome measures on patients who completed follow-up (per-protocol analysis).

	Baseline		1 Month		3 Months		6 Months		*p* Value
	OT	HA	OT	HA	OT	HA	OT	HA	
n	22	22	22	22	22	22	22	22	
WOMAC pain subscore	6.8 ± 2.7	7.3 ± 3.7	3.7 ± 3.1	2.8 ± 3.1	3.7 ± 2.7	2.9 ± 3.1	4.4 ± 3.1	3.9 ± 3.8	0.001
WOMAC total score	45.6 ± 15.1	45.8 ± 20.1	18.7 ± 14.3	14.0 ± 13.1	17.7 ± 14.0	14.8 ± 16.4	20.9 ± 15.8	17.9 ± 16.7	<0.001
KOOS	79.1 ± 24.9	72.2 ± 27.3	40.0 ± 26.2	32.4 ± 26.9	38.9 ± 28.1	30.9 ± 32.8	44.8 ± 30.5	35.8 ± 32.3	<0.001
NRS	5.6 ± 2.2	5.8 ± 1.7	2.3 ± 2.2	2.6 ± 2.2	2.9 ± 2.6	2.5 ± 2.3	3.6 ± 2.6	3.2 ± 2.7	<0.001

Continuous variables are expressed as means ± standard deviations; categorical data are expressed as counts (percentages). Abbreviations: OT: oxygen–ozone therapy; HA: hyaluronic acid; WOMAC: Western Ontario and McMaster Universities Osteoarthritis Index; KOOS: Knee injury and Osteoarthritis Outcome Score; NRS: Numeric Rating Scale.

**Table 3:** Between-group differences in the primary outcome.

	OT Group (Group A)	HA Group (Group B)	*p* Value
WOMAC LK 3.1 pain score (baseline)	6.8 ± 2.7	7.3 ± 3.7	0.521
WOMAC LK 3.1 pain score (1 month)	3.7 ± 3.1	2.8 ± 3.1	0.261
WOMAC LK 3.1 pain score (3 months)	3.7 ± 2.7	2.9 ± 3.1	0.345
WOMAC LK 3.1 pain score (6 months)	4.4 ± 3.1	3.9 ± 3.8	0.633

Continuous variables are expressed as means ± standard deviations; categorical data are expressed as counts (percentages). Abbreviations: OT: oxygen–ozone therapy; HA: hyaluronic acid; BMI: body mass index; WOMAC: Western Ontario and McMaster Universities Osteoarthritis Index.

**Table 4:** Between-group differences in the secondary outcomes.

	OT Group (Group A)	HA Group (Group B)	*p* Value
WOMAC total score (baseline)	45.6 ± 15.1	45.8 ± 20.1	0.888
WOMAC total score (1 month)	18.73 ± 14.3	14.04 ± 13.14	0.223
WOMAC total score (3 months)	17.69 ± 14.0	14.81 ± 16.4	0.500
WOMAC total score (6 months)	20.86 ± 15.8	17.86 ± 16.7	0.544
KOOS (baseline)	80.91 ± 21.0	73.13 ± 29.4	0.319
KOOS (1 month)	39.96 ± 26.2	32.42 ± 26.9	0.311
KOOS (3 months)	38.92 ± 28.1	30.92 ± 32.8	0.349
KOOS (6 months)	44.77 ± 30.5	35.77 ± 32.3	0.347
NRS (baseline)	5.81 ± 2.2	5.86 ± 1.6	0.939
NRS (1 month)	2.35 ± 2.2	2.58 ± 2.2	0.707
NRS (3 months)	2.92 ± 2.6	2.50 ± 2.3	0.538
NRS (6 months)	3.60 ± 2.6	3.23 ± 2.7	0.653

Continuous variables are expressed as means ± standard deviations; categorical data are expressed as counts (percentages). Abbreviations: OT: oxygen–ozone therapy; HA: hyaluronic acid; WOMAC: Western Ontario and McMaster Universities Osteoarthritis Index; KOOS: Knee injury and Osteoarthritis Outcome Score; NRS: Numeric Rating Scale.

## Data Availability

The dataset is available upon request to the corresponding author.

## Supplementary Materials

The following supporting information can be downloaded at: https://www.mdpi.com/article/10.3390/ijms24108788/s1.
