# Supplementary material for: Ozone Therapy versus Hyaluronic Acid Injections for Pain Relief in Patients with Knee Osteoarthritis: Preliminary Findings on Molecular and Clinical Outcomes from a Randomized Controlled Trial
Source: Int J Mol Sci. 2023 May 15;24(10):8788. doi: 10.3390/ijms24108788 (PMC10218112; doi:10.3390/ijms24108788)
Supplement: Supplementary file 1 [file ijms-24-08788-s001.zip › ijms-2316158-supplementary.pdf]

Table S1. Intra-group differences in outcome measures including all recruited patients (intention-to-treat analysis).

	n	**OT Group** (Group A)	n	**HA Group** (Group B)	**P value**
WOMAC LK 3.1 pain score (baseline)	26	7.0 ± 2.9	26	7.6 ± 3.6	0.521
WOMAC LK 3.1 pain score (1 month)	26	4.0 ± 3.2	26	4.0 ± 3.2	0.261
WOMAC LK 3.1 pain score (3 months)	26	4.0 ± 2.7	26	2.9 ± 2.5	0.345
WOMAC LK 3.1 pain score (6 months)	22	4.4 ± 3.1	22	3.9 ± 3.8	0.634
WOMAC total score (baseline)	26	46.5 ± 15.4	26	47.3 ± 20.5	0.963
WOMAC total score (1 month)	26	19.5 ± 15.3	26	14.6 ± 14.0	0.223
WOMAC total score (3 months)	26	18.5 ± 14.5	26	14.4 ± 15.6	0.500
WOMAC total score (6 months)	22	20.9 ± 15.8	22	17.9 ± 16.7	0.544
KOOS (baseline)	26	80.9 ± 21.0	26	73.1 ± 29.4	0.347
KOOS (1 month)	26	43.1 ± 26.2	26	33.8 ± 27.5	0.311
KOOS (3 month)	26	41.0 ± 28.1	26	30.3 ± 31.4	0.349
KOOS (6 months)	22	44.8 ± 30.5	22	35.8 ± 32.3	0.348
NRS (baseline)	26	5.8 ± 2.2	26	5.9 ± 1.6	0.781
NRS (1 month)	26	2.4 ± 2.4	26	2.5 ± 2.1	0.707
NRS (3 month)	26	2.9 ± 2.4	26	2.3 ± 2.2	0.538
NRS (6 months)	22	3.6 ± 2.6	22	3.2 ± 2.7	0.653
